# Survey of X-ray induced Cherenkov excited fluorophores with potential for human use

**DOI:** 10.1093/jrr/rrab055

**Published:** 2021-07-12

**Authors:** Arthur F Pétusseau, Petr Bruza, Brian W Pogue

**Affiliations:** Thayer School of Engineering, Dartmouth College, Hanover, NH 03755, USA; Thayer School of Engineering, Dartmouth College, Hanover, NH 03755, USA; Thayer School of Engineering, Dartmouth College, Hanover, NH 03755, USA

**Keywords:** X-ray induced molecular luminescence (XML), Cherenkov radiation, scintillation, therapeutic megavoltage X-rays, organic fluorophores

## Abstract

X-ray induced molecular luminescence (XML) is a phenomenon that can be utilized for clinical, deep-tissue functional imaging of tailored molecular probes. In this study, a survey of common or clinically approved fluorophores was carried out for their megavoltage X-ray induced excitation and emission characteristics. We find that direct scintillation effects and Cherenkov generation are two possible ways to cause these molecules’ excitation. To distinguish the contributions of each excitation mechanism, we exploited the dependency of Cherenkov radiation yield on X-ray energy. The probes were irradiated by constant dose of 6 MV and 18 MV X-ray radiation, and their relative emission intensities and spectra were quantified for each X-ray energy pair. From the ratios of XML, yield for 6 MV and 18 MV irradiation we found that the Cherenkov radiation dominated as an excitation mechanism, except for aluminum phthalocyanine, which exhibited substantial scintillation. The highest emission yields were detected from fluorescein, proflavin and aluminum phthalocyanine, in that order. XML yield was found to be affected by the emission quantum yield, overlap of the fluorescence excitation and Cherenkov emission spectra, scintillation yield. Considering all these factors and XML emission spectrum respective to tissue optical window, aluminum phthalocyanine offers the best XML yield for deep tissue use, while fluorescein and proflavine are most useful for subcutaneous or superficial use.

## INTRODUCTION

X-ray induced molecular luminescence (XML) signals can be used for molecular imaging in tissue, providing deep tissue sampling of relevant metabolic information if the probe is coupled to a targeting approach [1–3]. However, the origins of luminescence from organic molecules by X-ray excitation can be unclear at times, with the general phenomenon being referred to as radioluminescence. This can originate from different parts of the interaction cascade [4], often from secondary or tertiary radiation sources, being largely scattered electrons or low energy photons with high interaction cross sections. In general, organic molecules can be non-radiatively excited via scintillation mechanisms, or radiatively excited by Cherenkov radiation originating from high energy charged particles’ interaction with matter. However, the exact contribution of each mechanism is uncertain in most biologically relevant molecules. Yet, it is important to parse this out because the origin of the signal can affect the choice of X-ray energy and source used, as well as the efficiency of use. In this article we survey a set of molecular reporters with the goal to evaluate their potential as radioluminescent reporters, as well as to determine their primary mode of interaction with therapeutic (megavoltage) X-rays.

The choice of reporters tested here was guided by their having regulatory approval for human use as fluorescent agents or having been involved in human trials. The specific agents examined were fluorescein, methylene blue, proflavine, verteporfin, protoporphyrin IX, aluminum phthalocyanine. In an effort to examine direct scintillation further, additional metallophthalocyanines were included that might have high interaction cross section with X-rays, such as cobalt phthalocyanine and gadolinium phthalocyanine. In this survey their potential as radioluminescent reporters was quantified for emission yield, and secondarily their emission yield energy dependence was used to assay their primary mode of interaction with the X-rays. Scintillation is a direct linear reporter of dose, independent of energy in most cases of high energy MV photons, while changes in the X-ray energy spectrum from 6 MV to 18 MV [5] increases Cherenkov light yield by nearly a factor of two. The ratio of XML light yield per unit dose or per unit Cherenkov intensity was used as an assay to discern if the origins of radioluminescence from each molecule was Cherenkov mediated or scintillation mediated. Parsing out the emission origins can be challenging because of solvent radioluminescence background, which is largely Cherenkov light. Analysis of XML emission spectra was performed to separate the Cherenkov solvent background from the absorption and fluorescence of the probes. Excitation origins of the probes were evaluated looking at the 18 MV/6 MV signal ratios around their emission wavelength relative to the ratio of a scintillator and 1× phosphate buffered saline solution (PBS). This data should provide a useful way to determine which clinical or *in vivo* compatible tracers could be good radioluminescent probes for XML.

## METHODS

### Samples

The fluorophores considered in this survey are as follows: Fluorescein sodium salt (Fluorescein, Fluka, St Louis, MO), Proflavine hemisulfate salt hydrate (Proflavine, Sigma-Aldrich, St Louis, MO), Al(III) Phthalocyanine Chloride Tetrasulfonic Acid (AlPcS_4_, Frontier Scientific, Logan, UT), Co(II) Phthalocyanine Tetrasulfonic Acid (CoPcS_4_, Frontier Scientific, Logan, UT), Gd(III) Phthalocyanine tetrasulfonate tetrasodium salt hydroxide (GdPcS_4_, Frontier Scientific, Logan, UT), Protoporphyrin IX (PpIX, Sigma-Aldrich, St Louis, MO), Verteporfin (Sigma-Aldrich, St Louis, MO), Methylene blue (MB, Sigma-Aldrich, St Louis, MO).

The influence of different solvents on the molecules fluorescent efficiency was studied testing three different solvents, these being PBS (DPBS 1X, Corning Inc., Corning, NY), Dimethyl sulfoxide (DMSO) (Sigma-Aldrich, St Louis, MO) and Methanol (Fisher Chemicals, Waltham, MA). In this first step, all probes were tested in each of the solvents at 10 μM concentration.

In subsequent studies, all fluorophores were prepared in PBS solution. Both Verteporfin and Protoporphyrin IX (PpIX) were pre-dissolved in DMSO with a ratio of 0.1 mg/mL and thereafter dissolved in PBS at low concentrations as listed. The concentrations used for each probe in latter studies was chosen according to the initial results where maximal emission yield was found.

### Irradiation

**Fig. 1.**
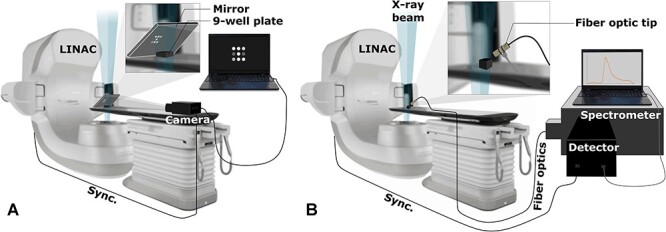
(A) Wide field imaging of nine-well plate; and (B) spectrally resolved imaging experimental setup. The X-ray beam is delivered from the bottom and the signal is acquired from the top using respectively a widefield camera coupled with a mirror and a fiber optic hold above the sample.

All samples were irradiated using a Varian TrueBeam (Varian Medical Systems, Palo Alto, CA) linear accelerator. A 10×10 cm^2^ photon beam was used, the energy being either 6 MV or 18 MV. For each energy, an extra thickness (1 cm and 3 cm, respectively) of Solid Water plastic (Sun Nuclear, Melbourne, FL) was added underneath the sample such that each sample was located at the maximum dose deposition depth [6]. All samples were irradiated from the bottom and imaged from the top in an attempt to reduce X-ray noise on the detector while maximizing the signal as shown in Fig. 1.

For each energy comparison, the dose delivered to samples was monitored using radiochromic film dosimeters (EBT-XD, Ashland, Bridgewater, NJ) in order to confirm that the same dose was delivered between 6 MV and 18 MV. The dose measured for 6 MV and 18 MV was 522 ± 3 cGy and 523 ± 3 cGy respectively.

### Intensity and spectroscopy

In order to explore Cherenkov and scintillations’ contribution in the luminescent processes, an intensified charge-coupled device (ICCD) camera (PiMAX3, Princeton Instruments, Acton, MA) was coupled to a SpectraPro 2300i spectrograph (Acton Research Corporation, Princeton Instruments) to capture luminescence emission spectra (Cherenkov vs scintillation assay). The spectrometer was calibrated for its spectral detection sensitivity. A 10 m fiber optic bundle was used to couple the sample emission to spectrometer, which was located outside the shielded medical linear accelerator vault in order to minimize stray X-ray noise effects on ICCD. The end of the fiber was placed above an 8 mL well with an angle of approximately 30° with respect to the solution surface (see Fig. 1b). Since the ionizing beam induced luminescence effect in the fiber [5], the sample was placed at the edge of the 10×10 cm^2^ beam in order to minimize the interaction between the photon beam and the fiber tip. For each probe, 600 monitor units (MU) were delivered at a rate of 600 MU/min. To ensure correct spectral sensitivity calibration that includes the fiber and other experimental conditions, additional correction steps were performed. The persistent X-ray induced scintillation signal from the fiber was subtracted from the raw data for each energy. Further, we used a known ~1/*λ*^2^ proportionality of emitted Cherenkov photons per wavelength *λ* of a pure Cherenkov emitter [7], in our case pure PBS. The detected emission spectrum of pure PBS under 18 MV irradiation exhibited a good match to the theoretical profile in the spectral range of 580–750 nm, but it deviated below 580 nm due to limited signal-to-noise ratio of the ICCD detector. We therefore created a spectral correction profile *k*(*λ*) by fitting a *k*(*λ*) *· 1*/*λ*^2^ model to the measured PBS spectrum in the 580–750 nm spectral range using an iterative non-linear least square method (Matlab R2018a, The MathWorks Inc., Natick, MA), and by taking the ratio of measured spectra to the fitted model in the full spectral range of 450–750 nm. This calibration was then applied to all fluorescence spectra acquired. Finally, a plastic scintillator EJ-212 (Eljen, Windsor, CT) was measured to serve as a control of pure scintillator response, assuming Cherenkov light is negligible to the large scintillation yield of EJ-212.

For quantitative analysis of the probes’ integrated emission spectra (concentration dependence study), the fluorophores at their ideal concentrations were irradiated and their luminescence emissions were imaged in a black nine-well plate using a red-sensitive PiMAX4 (Princeton Instruments, Acton, MA) intensified CCD camera. For each probe, solutions were made at zero, 10 nM, 100 nM, 500 nM, 1 μM, 5 μM and 10 μM. In addition, data points were added for proflavine at 50 μM and for fluorescein at 50 μM, 100 μM and 500 μM. 2 mL (corresponding to 2 cm thickness) aliquots were transferred to 2 cm deep wells made of black high-density polyethylene, positioned on top of Solid Water plastic slab. In this setup, the samples were irradiated from below (Gantry angle of 180°) and imaged using a front-surface mirror making a 45° angle with the upper surface of the well plate (see Fig. 1a). This configuration allowed for minimization of the X-ray noise on the CCD sensor. The intensifier pulses of 4 μs width were synchronized with the linear accelerator pulses. Each acquisition was made over 600 MU at a rate of 600 MU/min. All acquired frames were thereafter summed to obtain a total number of photons detected over the whole dose delivery. The exact same setup was used for sample irradiation in the solvent effect study.

**Fig. 2.**
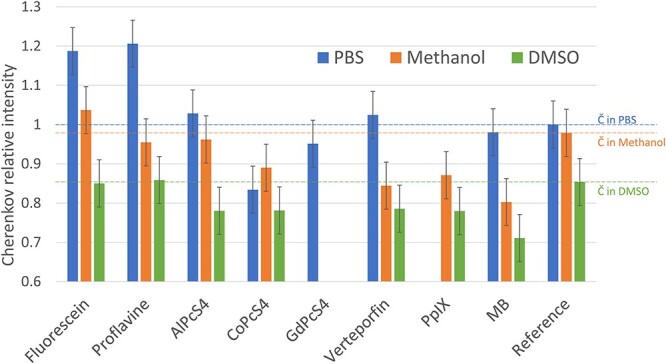
Luminescent signal from nine organic molecules dissolved in PBS (blue), methanol (orange) and DMSO (green), irradiated by an 18 MV X-ray beam. The signals are normalized to the Cherenkov signal in PBS.

**Fig. 3.**
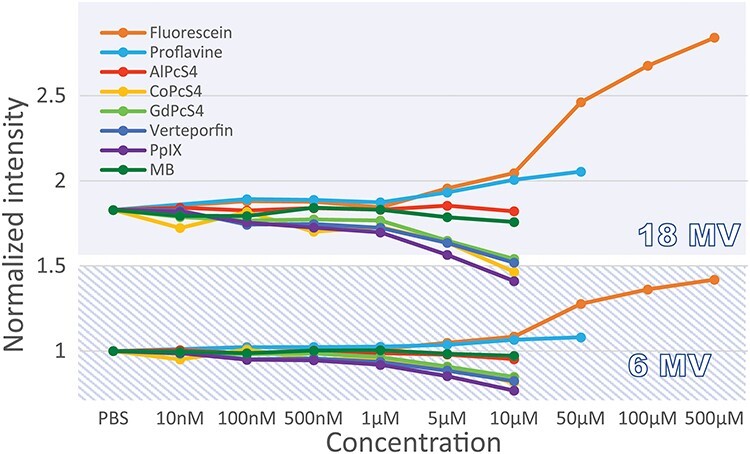
Comparison of the radioluminescence from nine probes in PBS solution exposed to 18 MV and 6 MV X-ray beams, depending on concentration. The intensity is normalized to the Cherenkov level in PBS corresponding to 6 MV irradiation.

For both spectrally resolved data and wide field data, the measurement error was calculated using a set of five measurements in the same conditions. This error was then added to the single measurement data from Figs 2,3 and 5.

## RESULTS

### Solvent effects

It has been shown that X-ray excited luminescence properties of molecules can be altered by a solvent [8]. Here we studied the influence of three different solvents on relative XML intensity. Fig. 2 shows the spectrally-unresolved XML intensities detected in red spectral range detected from 10 μM fluorophore solutions and pure solvents irradiated by 18 MV photon beam. The missing data corresponds to cases where the contrast agent was insoluble in the solvent. All probes except CoPc4 exhibit the brightest luminescence when dissolved in PBS, yet only fluorescein, proflavine, AlPcS4, and Verteporfin showed signals distinctly above the Cherenkov level. In the case of Verteporfin, the slightly superior signal was within the accuracy level of this measurement. Despite the fact that the Cherenkov signal is almost as important in methanol as in PBS, we observed that this solvent quenched the fluorescence of all probes. The level of this quenching importance varied with the probe. Only fluorescein exhibited an emission signal above the Cherenkov signal in methanol. Finally, no probe showed a higher signal than the Cherenkov signal in DMSO. While Cherenkov intensity is far lower in DMSO than in PBS and methanol, DMSO also showed strong quenching for most of the probes. Fluorescence emission quantum yields, maximum excitation and emission wavelengths, fluorescent lifetime and solubility of each agents are shown in Table 1 for comparison.

**Table 1:** Organic fluorescent probes characteristics. Fluorescence quantum yield (QY), maximum excitation/emission wavelength, lifetime and solubility corresponding to each probe listed in Fig. 2 [8–19]

Probe	QY	λex (nm)	λem (nm)	Lifetime (ns)	Solubility
Fluorescein	79%	494	521	4	PBS
Proflavine	44%	444	515	4	PBS
AIPcS_4_	40%	673	680	5	PBS
GdPcS_4_	-	636	688	-	PBS
CoPcS_4_	-	669	-	-	PBS
Verteporfin	5%	436	692	6	DMSO
PpIX	9%	405	634	16	DMSO
MB	5%	664	684	<1	PBS

### Concentration dependence

To find the maximum of fluorescence emission, the spectra were measured at different probe concentrations when irradiated by both 6 MV and 18 MV X-rays. Fig. 3 shows light intensity normalized to 6 MV Cherenkov in PBS, as a function of concentration for all probes listed above. The dotted lines correspond to the 6 MV photon beam while the solid lines represent the 18 MV data. For each probe, the intensity level was compared to the luminescence coming from PBS, which corresponds to Cherenkov light only. In the case of Fluorescein, Proflavine and AlPcS_4_, light emission increased when a certain concentration was reached and then saturated at higher concentrations. For all probes, we observe that higher concentration involved a loss of luminescent signal, presumably due to the effects of probe absorption of the Cherenkov light of the medium. The ratio of intensity between 6 MV and 18 MV for PBS is 1.83 ± 0.06, which corresponds to the expected ratio for Cherenkov [20].

### Cherenkov vs scintillation assay

**Fig. 4.**
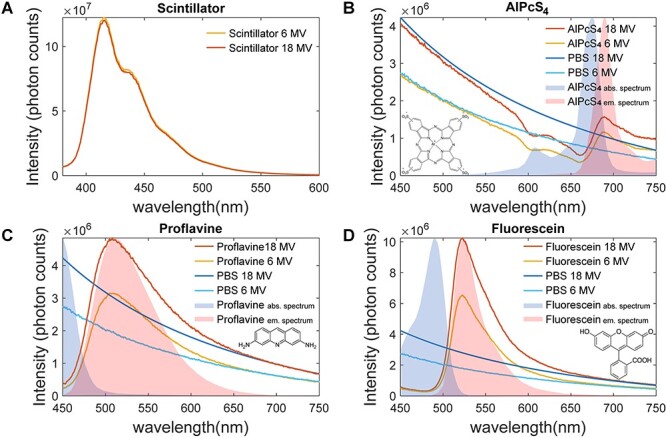
Comparison of 18 MV and 6 MV emission spectra for: (A) scintillator at a fixed dose level; and those for (B) AlPcS_4_; (C) Proflavine; and (D) Fluorescein in PBS. Each organic molecule spectra is overlapped with 18 MV and 6 MV PBS emission spectra as well as the molecules visible light absorption and emission spectra. Inset in each graph is its molecular bond diagram.

**Fig. 5.**
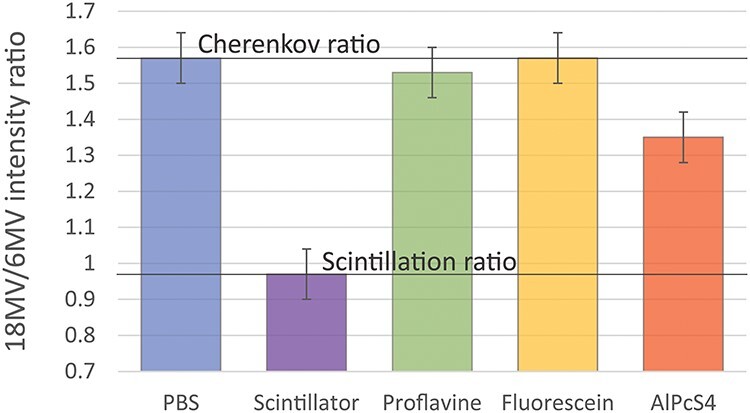
Ratios of 18 MV to 6 MV signals from pure PBS, a scintillator, proflavine, fluorescein and AlPcS_4_. The ratio for PBS Cherenkov, 1.57 ± 0.07, is normalized by the ratio coming from the scintillator equals 0.97 ± 0.07. The organic molecules show ratios in between, depending on the influence of each phenomenon.

Fig. 4 shows the emission spectra of an EJ-212 scintillator, AlPcS_4_, proflavine and fluorescein when exposed to 6 MV and 18 MV X-ray beams. The spectra of molecular probes also include Cherenkov emission spectra of PBS for each used X-ray energy. Each figure is overlayed with visible light absorption and emission spectra (acquired respectively with Cary 50 Bio UV–Visible Spectrophotometer, Agilent Technologies, Santa Clara, CA and Fluoromax 4, Horiba, Kyoto, Japan) for comparison purposes. The difference of signal between the two energies is shown by Fig. 5 which represents the ratio of light intensity around the fluorophores’ maximum emission wavelength (± 10 nm) between the 18 MV and 6 MV signal. Again, on these graphs, the ratio corresponding to PBS is added for comparison.

The coincidence of the two curves in the scintillator graph (Fig. 4 [A]) indicates that there is no change of light emission as the energy varies. This is highlighted by the corresponding constant ratio of 0.97 ± 0.07 on Fig. 5. However, this is not true for the three organic molecules which show a different luminescence intensity as the X-ray beam energy varies. In the case of proflavine and fluorescein, we measured ratios of 1.53 ± 0.07 and 1.57 ± 0.07, respectively, which are similar to the ratio of Cherenkov emission intensities from PBS. On the other hand, AlPcS_4_ is showing an inconsistency in its ratio which goes down to approximately 1.35 ± 0.07 around its emission peak.

Finally looking at X-ray emission spectra, we see that many probes exhibit luminescence decreases in comparison to the PBS spectra. These correspond to the probes’ absorption spectra. Around their maximum emission wavelength, CoPcS_4_, GdPcS_4_, Verteporfin, PPIX and MB show weaker signal than PBS due to their low quantum yield. The data corresponding to the later molecules is therefore not shown in Figs 4 and 5.

## DISCUSSION

### Solvent effect

It has been shown in Fig. 2 that when exposed to high energy X-rays, PBS, DMSO and Methanol’s light emission varied depending on the solvent. This is due to fluorescence solvent quenching, a complex phenomenon with many causes, of which the major ones are solvent polarity and viscosity [21]. When combined with an inadequate solvent, fluorophores can aggregate leading to quenching of their fluorescence through a phenomenon called aggregation-caused quenching [22]. This quenching effect is particularly true for DMSO and Methanol, which is not surprising given that most of these molecules have been designed to be soluble in an aqueous biological environment.

### Concentration dependence

In order to be relevant for human use, contrast agents must show strong signal at tolerable concentrations as governed by FDA guidelines [23]. In the case of X-ray or Cherenkov excited fluorescence, a probe must have an emission signal greater than the Cherenkov baseline signal. Fluorescence of all probes at low concentrations (10 nM – 1 μM) went undetected for both spectrally integrated and resolved experiments. In the broadband detection scheme (Fig. 3), the intensities are similar to the Cherenkov emission intensity of pure PBS, exhibiting a ratio of 1.83 ± 0.06 between 18 MV and 6 MV energies. As the concentration increases, fluorescein, proflavine and AlPcS_4_ show increasing fluorescence until a certain point where the signal saturates (data cropped on Fig. 3). This is attributed to fluorescence self-quenching [24] of the molecules within the solution. As for the case of proflavine, fluorescein and AlPcS_4,_ the concentrations exhibiting the brightest luminescence are 50 μM, 500 μM and 10 μM, respectively. In spectrally-resolved data (Fig. 4), we further show that the emission exceeds Cherenkov intensity level also in AlPcS_4_ around its emission peak.

### Cherenkov vs scintillation

The mechanisms behind organic molecules excitation during irradiation are not always clear because of the series of radiative and non-radiative events that occur. The two major pathways these molecules could be excited by are considered here, with the first being Cherenkov light generated throughout the medium upon x-ray irradiation. This broadband light emitted due to secondary electrons during irradiation can in principle excite any of the used probes, although the efficiency of Cherenkov production is fairly low. Importantly, Cherenkov intensity depends on both the X-ray energy spectrum, as well as the total dose deposited [25]. The second way radioluminescence can be excited is through scintillation mechanisms, that are largely mediated by excitation of the solvent, followed by non-radiative energy transfer to the emitter molecule [7]. In contrast with Cherenkov, scintillation is known to be energy independent throughout this high energy MV X-ray range [5]. Thus, by irradiating each sample with different energies (6 MV vs 18 MV) while keeping the deposited dose constant, we expect to be able to quantify the contribution from both phenomena.

For the three probes shown in Fig. 4, the light intensity is higher than the Cherenkov intensity around their emission peak. At the same time, it can be seen that the Cherenkov signal is absorbed by probes in their absorption spectral band. Those probes that have the highest luminescence essentially are redistributing the UV and blue Cherenkov photons from higher energies to lower energy photons via fluorescence. Note that the concentration of each probe was chosen to be optimal for emission intensity within PBS. Interestingly, as seen in Fig. 5, the AlPcS_4_ probe exhibited a mixed scintillation (63%) and Cherenkov-excited fluorescence (37%) behavior.

The difference in PBS Cherenkov emission ratio as seen in Figs 3 and 4 (1.83 ± 0.06 versus 1.57 ± 0.07, respectively) is most probably due to the acquisition setup. Data in Fig. 3 was acquired with a large numerical aperture lens while the spectrally resolved data were acquired using a fiber optics coupling. Given that Cherenkov emission is more forward directed as the exciting particles’ energy increases [26], it is possible that less light was captured with the fiber optics for 18 MV than for 6 MV due to its small numerical aperture. Nonetheless, these ratiometric values of fluorophores’ emission relative to PBS emission can be used in a fixed setup geometry as a measure of the scintillation versus Cherenkov excitation of the probes since the same changes apply to all studied samples.

### CONCLUSION

The aim of this work was to identify the excitation mechanisms of various X-ray excitable fluorescent molecules. The choice of these tracers was specifically made based on their approval for human use as fluorescent agents. These findings may be utilized in future studies of *in vivo* X-ray excited luminescence [27] or in dosimetry applications [28]. The choice of these tracers was specifically made based on their approval for human use as fluorescent agents. The excitation of fluorescent organic molecules is mainly Cherenkov mediated, even though some of them, particularly aluminium phthalocyanine, seem to luminesce through scintillation mechanisms also. Fluorescein and proflavine show strong signals to Cherenkov ratios even at low concentrations, although the emission is still largely in the blue–green part of the spectrum. However, AlPcS_4_ shows a more interesting signal in the red spectrum, consistent with the diffuse light transport window of tissue [29,30]. Thus, radioluminescence reporting from fluorescein and proflavine are ideal when blue–green emission is desired and high quantum yield phthalocyanines are likely optimal when red to near-infrared emission is desired. Further exploration of the potential for direct scintillation in tissue from molecular probes that have similar interactions with their environment as AlPcS_4_ may be of further benefit.
